# Tackling Thyroid Cancer in Europe—The Challenges and Opportunities

**DOI:** 10.3390/healthcare10091621

**Published:** 2022-08-25

**Authors:** Denis Horgan, Dagmar Führer-Sakel, Paula Soares, Clara V. Alvarez, Laura Fugazzola, Romana T. Netea-Maier, Barbara Jarzab, Marta Kozaric, Beate Bartes, James Schuster-Bruce, Luigino Dal Maso, Martin Schlumberger, Furio Pacini

**Affiliations:** 1 European Alliance for Personalised Medicine, 1040 Brussels, Belgium; 2 Department of Endocrinology, Diabetes and Metabolism, Endocrine Tumour Center at West German Cancer Center, University Hospital Essen, University of Duisburg-Essen, 45122 Duisburg, Germany; 3 Instituto de Inovação e Investigação em Saúde/Institute of Molecular Pathology and Immunology of University of Porto (I3S/IPATIMUP), 4200-465 Porto, Portugal; 4 Faculty of Medicine of the University of Porto, 4200-319 Porto, Portugal; 5 Neoplasia & Endocrine Differentiation, Centro de Investigación en Medicina Molecular y Enfermedades Crónicas (CIMUS), University of Santiago de Compostela (USC), 15782 Santiago de Compostela, Spain; 6 Instituto de Investigacion Sanitaria (IDIS), 15706 Santiago de Compostela, Spain; 7 Division of Endocrine and Metabolic Diseases, IRCCS Istituto Auxologico Italiano, 20145 Milan, Italy; 8 Department of Pathophysiology and Transplantation, University of Milan, 20122 Milan, Italy; 9 Division of Endocrinology, Department of Internal Medicine, Radboud University Medical Center, 6525 GA Nijmegen, The Netherlands; 10 Department of Nuclear Medicine and Endocrine Oncology, Maria Sklodowska-Curie National Research Institute of Oncology Gliwice Branch, 44-102 Gliwice, Poland; 11 Association Vivre sans Thyroïde, 31490 Léguevin, France; 12 Department of Otolaryngology, Saint George’s University Hospitals NHS Foundation Trust, London SW17 0QT, UK; 13 Cancer Epidemiology Unit, Centro di Riferimento Oncologico di Aviano (CRO), 33081 Aviano, Italy; 14 Department of Nuclear Medicine and Endocrine Oncology, Institut Gustave Roussy, Université Paris Saclay, 94805 Villejuif, France; 15 Section of Endocrinology, University of Siena, 53100 Siena, Italy

**Keywords:** thyroid cancer, rare cancer, personalised medicine, challenges, opportunities, treatment, diagnosis, policy framework

## Abstract

Thyroid cancer (TC) is the most common malignancy of the endocrine system that affects the thyroid gland. It is usually treatable and, in most cases, curable. The central issues are how to improve knowledge on TC, to accurately identify cases at an early stage that can benefit from effective intervention, optimise therapy, and reduce the risk of overdiagnosis and unnecessary treatment. Questions remain about management, about treating all patients in referral centres, and about which treatment should be proposed to any individual patient and how this can be optimised. The European Alliance for Personalised Medicine (EAPM) hosted an expert panel discussion to elucidate some of the challenges, and to identify possible steps towards effective responses at the EU and member state level, particularly in the context of the opportunities in the European Union’s evolving initiatives—notably its Beating Cancer Plan, its Cancer Mission, and its research funding programmes. Recommendations emerging from the panel focus on improved infrastructure and funding, and on promoting multi-stakeholder collaboration between national and European initiatives to complement, support, and mutually reinforce efforts to improve patient care.

## 1. Introduction

Thyroid cancer (TC) is a type of cancer which is usually treatable and, in most cases, curable, but can sometimes recur after treatment [1]. Despite advances in understanding of the disease, uncertainties still cloud screening, diagnosis and treatment—uncertainties that have grown with the increasing sophistication of tools, such as refined ultrasound and molecular testing, and widening choices of therapy [2].

Increasing incidence, stable mortality

Multiple sources suggest that TC incidence is increasing, with Lim et al. [3] noting a rise of 3% annually in the United States between 1974 and 2013, and Smittenaar et al. [4] finding it is the fastest accelerating cancer (males: 2.49%, females: 2.34%) in average annual percentage change. In 2020, there were 570,000 new cases at the global level, with 77% in women, and 78,000 cases and 7000 deaths in Europe [5]. Overdiagnosis alone is not a sufficient explanation for the increases, according to Smittenaar et al., 2016, who note small projected increases in age-standardised rates (ASR) mortality for women.

Meanwhile, mortality rates are unchanged or even decreasing. The American Cancer Society reported an increase of 0.6 % per year between 2009 and 2018, but stabilisation has since been observed [6]. According to the International Agency for Research on Cancer, there is no correlation between incidence and mortality [7]. However, survival rates decrease with the advancement of the cancer stage [8].

Multiple manifestations

The disease manifests either as differentiated thyroid cancer (DTC), poorly differentiated thyroid cancer (PDTC), or undifferentiated or anaplastic thyroid cancer (ATC)—all of which are derived from thyroid follicular cells—or medullary thyroid cancer (MTC), which is derived from non-follicular calcitonin + cells [9]. DTC has three basic subtypes: papillary thyroid cancer (PTC) (80% of all thyroid cancer cases), follicular thyroid cancer (FTC), and Oncocytic Thyroid Carcinoma (OTC) [10]. Caught early, TC—excluding anaplastic TC—is one of the most treatable forms of cancer.

Treatment options

Treatment options and outcomes vary across these subtypes, with advanced TC being a rare tumour benefiting from only limited recent advances. Several genomic alterations are relevant across the different histological types of TCs [11]. Identifying the most appropriate therapy is challenging, since somatic testing is insufficiently standardised, with only a few actionable biomarkers [11,12]. Much of the debate revolves around effective early detection and treatment [13,14] as the paradigm for management has changed over decades [15]. A potentially cancerous lump revealed by an ultrasound scan of the neck can lead to a confirmed diagnosis with biopsy, at which point CT and MRI scans are needed to check for metastasis [1]. However, overdiagnosis can lead to overtreatment with unnecessary thyroidectomies, and a substantial and sustained increase in incidence without accompanying increase in mortality could serve as indirect evidence of overdiagnosis and resulting overtreatment [16]. 

Initial treatment options of surgery, radioiodine remnant ablation and/or thyroid hormone replacement treatment were accompanied in the pre-ultrasound era by a long- lasting debate on whether lobectomy was outperformed by bilateral lobectomy, or whether surgery had any effect at all on patient survival [17]. From 1980, improved knowledge of the biology of DTC was complemented by newer diagnostic tools and advances in therapy—including recombinant human TSH, applications of radioligands for imaging and therapy—and tailored and risk-adapted methodologies, the use of radioiodine, and thyroid hormone treatment [18], and pathological techniques improved, with thyroglobulin (basal and stimulated) offering a marker for any thyroid cell [19]. 

With screening, reported incidence increased, and treatment strategy changed accordingly, raising costs and the clinical burden. Neck ultrasounds and fine needle aspiration (FNA) increased sharply, and thyroidectomies for benign conditions more than doubled in some countries [20]. Treatment costs tripled in the USA after 1975, from 4.9 to 14.3/100,000 [21]. However, this brought no guarantee of improvement in overall health; it permitted detection of minimal disease, and much of the increase was due to microcarcinomas. Currently, microcarcinomas (tumors < 1 cm) are the most common TC diagnosed worldwide, either through sensitive ultrasound or incidentally in the study of thyroid pathology pieces after benign surgeries (nodular goiter, adenomas). New nomenclature groups these microcarcinomas into Papillary MicroTumour (PMT) and Papillary MicroCarcinoma (PMC) [22]. Most are indolent, but some lead to advanced thyroid carcinomas with metastasis and death. A “benign” or “negative for malignancy” result is obtained from 60 to 70% of all thyroid fine needle aspiration biopsies (FNABs), avoiding unnecessary surgery. Initial sonographic assessment of thyroid nodules can distinguish benign nodules from those with suspicious or malignant features requiring further management, including FNAB, some auxiliary molecular techniques and thyroid surgery [23]. There is a need for optimised surveillance based on risk of recurrence and death for the increased number of TC survivors, since a small proportion develop significant recurrence requiring further treatment [24]. 

The release of Iodine-131 in the Chernobyl accident also led to increased incidence of TC (and other thyroid diseases) [25], including childhood TC in Belarus after only 4 years, and which continued to grow [26]. The number of operated TCs was highest in younger age groups, mostly 4–10 years after the incident, so later increased numbers in older age groups are likely to be more an effect of screening [27]. 

A roundtable organised by the European Alliance for Personalised Medicine (EAPM) at the end of March, 2022, assessed the challenges relating both to the demand side (governance, clinical trials framework and funding, clinical standardisation, and awareness and education) and to the supply side (reimbursement, infrastructure for conducting and validating tests, and testing access driven by evidence generation) to improve diagnosis and treatment of patients with TC.

## 2. Materials and Methods

A literature review was conducted to characterise specific challenges and opportunities related to thyroid cancer diagnosis and treatments across European countries. This is supplemented with different expert inputs gained at the roundtable organised by EAPM at the end of March, 2022. Attendees were drawn from key stakeholders whose interaction created a cross-sectoral, highly relevant, and dynamic discussion forum. Around 15 participants were involved and included public health decision makers, representatives from the European Commission, Members of the European Parliament, patient organisations and umbrella organisations representing interest groups and associations actively engaged in the field. 

## 3. Results

Being a type of cancer with a usually good prognosis, but with a relevant proportion of cases that can progress and result in patient death, TC still lacks established strategies that help physicians and patients to face the disease. Several issues and gaps in the management come from experts’ opinions and a review of the literature. These are presented below, grouped according to the following categories: challenges, divergences, and national perspectives.

### 3.1. The Challenges

On the demand side (Table 1), governance is lacking, in that international guidelines [28,29] are not completely concordant, and there are few European recommendations from ETA for specialised cases, such as the management of pediatric TC. TC is managed by endocrinologists, while oncologists mainly play a role in managing ATC or late-stage Advanced TC. The European Thyroid Association (ETA) has reached a consensus and produced guidelines regarding it [30]. Guidelines are also published by the European Society for Medical Oncology (ESMO) and the most recent were published in April 2022 [29]. 

An adequate clinical trial framework (and appropriate funding) does not exist. Only limited research has been performed on combined treatments such as check-point inhibitors and tyrosine kinase inhibitors (TKIs) [31,32]. In addition, EU data protection rules impose limits on transatlantic data exchange, which inhibits joint academic trials.

Clinical standardisation is far from sufficient. Partly because of uncertainties over treatment and partly because of differing morbidity patterns and the divergences among national health systems and medical regulations, there are still wide variations across Europe in the approach to TC, including the heterogeneity among TC treaters and even from region to region within individual states. Different guidelines exist, but with little consensus and compliance at the national level, and less still at the international level. For ATC, no approved and registered pharmacological strategies exist for mechanism-based combination treatments.

Awareness of TC diagnosis and management is low, as for rare cancers in general, with gaps in clinicians’ awareness of such guidelines, and public awareness is very low [33]. There are few effective programmes of education in place to remedy these deficiencies. Knowledge on the relationship between drug efficacy and the molecular profile of individual patients is limited. The optimal timepoint of starting therapy with TKIs has been established because specific indications have to be given regarding when to start the therapy and also the continuation of therapy must be done until progression or interruption happened due to adverse events [29,34].

At the heart of the problem is the lack of knowledge on TC. The disparate guidelines and other similar recommendations are not used in clinical practice, because the level of evidence is uneven and often very low, based on small and often biased retrospective studies. Many clinicians are resistant to changing their practice on the basis of the questionable data available. Prospective trials are urgently needed to provide convincing data, with EU funding needed to support academic trials and close engagement of scientific societies and expert centres.

On the supply side (Table 1), reimbursement is patchy, uncertain, and inadequate across the EU, both for diagnostics (and particularly advanced diagnostics) and for therapeutics. 

In DTC, around 80% of all cases would harbor actionable alterations such as BRAF, RET, mTOR or NTRK. Even if not all products are European Medicines Agency (EMA)-approved, TC emerges as a third cancer type where testing is valuable after NSCLC and CRC [35,36,37,38,39,40,41].

In order to optimise patient outcomes, access to genomic profiling needs to increase; however, the testing methodology and the infrastructure for conducting and validating tests is uneven at the EU level, unstandardised, or inadequately quality-controlled. 

Furthermore, guidelines mainly focus on clinically actionable biomarkers with approved therapies.

Of the various methodologies used to detect genomic alterations, Next-Generation Sequencing (NGS) gives the most comprehensive view across a large number of genes, identifying mutations, fusions, and other alterations, using a minimal quantity of tissue. However, this is not yet routine in clinical practice and, for ATC NGS, is not fast enough, so a new methodology should be implemented. There is insufficient provision of molecular profiling and precision medicine-based selection for patients with aggressive tumors. All TC patients should be tested for all potential actionable biomarkers when they reach the advanced stage before treatment decision. However, for that to happen, all tests should become available and reimbursed on approval of the targeted treatment, with guidelines updated and implemented accordingly. 

Rising incidence rates have led to some less aggressive clinical management, but data are still insufficient to provide confidence in decisions over treatment. Screening for TC remains uneven and should be discouraged [42]. There is also a problem with the burden of unregistered cases. 

There is insufficient provision of multidisciplinary forums to ensure collaboration among the wide range of stakeholders involved, ranging from supervising doctors, primary care physicians, surgeons (general, endocrine, or ENT), pathologists (general or specialised), endocrinologists, and nuclear medicine physicians to oncologists. Furthermore, patients with TC ideally should discuss the role of genomic-biomarker testing for the management of their cancer with their treating physician [13].

Testing access is driven less by evidence generation than by local circumstances and conditions. Testing access is also impacted by the variations in diagnostic laboratory coverage, driven by either insufficient infrastructure or availability of test technologies, but also by inappropriate referral pathways [43]. Patient access to appropriate and standardised diagnostic tests, therapies, and expertise varies across Europe [44,45].

### 3.2. Divergences

As a result, there are too many diagnoses of clinically irrelevant TC conducted from country to country, often incomplete, leading to overtreatment and non-specialist treatment, while underdiagnosis adversely affects care in rare and aggressive TCs. Management of care is uneven. In addition, the availability of therapies—particularly pharmaceutical—differs from country to country, and even average waiting times for treatment vary, leading to unequal quality or access to treatment across Europe.

Factors for variance include availability of tests, different diagnostic pressures, and national approaches to commissioning. Access to and implementation of molecular genotyping is an issue across Europe. A European survey [46] of molecular genotyping in refractory TCs among 86 practitioners in 18 European countries suggested a range of genotyping methods, mainly using DNA-based techniques, but also with significant use of RNA-based techniques and immunohistochemistry. The reasons for not prescribing tumor genotyping varied from non-reimbursement to lack of established workflow or lack of access to targeted therapy or a judgment that was not suitable for the patient. An institutional molecular tumor board was available in a minority of situations and routine access to selective therapies occurred in 40% of cases [46]. 

An EORTC/EAPM survey [47] of 94 institutions from 20 countries revealed a wide diversity in the number of patients evaluated per year, in molecular testing frequency and methodology, widespread conditions on prescription, and highly specific conditions for the offer of TKI and immunotherapy. 

### 3.3. National Perspectives

An illustrative snapshot of some of the national perspectives (Table 2)—and particular challenges—emerged through the panel discussion, as follows:

In Denmark, where a rapidly growing incidence is caused almost exclusively by low-mortality papillary tumors, accurate diagnostic methods are increasingly important to deal with the growing number of patients. However, preoperative assessment of thyroid nodules remains a diagnostic challenge. Scintigraphy and ultrasound are performed as part of the investigation, and cold nodules on scintigraphy and suspicious nodules on ultrasound are further investigated with FNA [48].

France has one of the highest incidence rates in Europe, with TC becoming the third most commonly diagnosed cancer in women. The rapid increases in incidence rates were driven by the papillary subtype, whereas, conversely, the incidence rates of anaplastic cancers demonstrated decreasing trends. There was also an epidemic of microcarcinomas (43% of operated cancers, period 1998–2001) associated with the extensiveness of thyroidectomies. Multicentric studies showed a significant increase, from 1980 to 2000, in ultrasonographic (3 to 84.8%) and cytological procedures (8 to 36% of patients with thyroid nodules), as well as a significant association between the increase in TC prevalence among operated patients (12.5 to 37%) and the spread of fine needle aspiration [49]. The magnitude of overdiagnosis increased rapidly and varied substantially across regions [50]. A National Refractory Thyroid Cancer Tumor Board created in 2008 brings together a national network of 30 expert treatment centres, to allow each patient with locally advanced or metastatic TC to access optimal management and therapeutic innovation. Its virtual tumor board meets every 2 weeks. It operates a national database for refractory TC and conducts or sponsors clinical trials. The patient perspective is taken account of via Vivre sans Thyroïde, an online patient forum created in 2000 that receives some 4000 visitors a day and provides evidence-based information, translation of medical terms, and exchanges of experience [51]. It ensures close cooperation with doctors, associations, and umbrella organisations. Its aims are to help patients to be correctly informed, avoiding overdiagnosis and overtreatment, but also underdiagnosis and undertreatment.

In Germany, cases have increased in part due to a rise in well-differentiated PTC [52]. There is good cost-free access of all patients to all levels of TC care, with virtually unrestricted access for all EMA-approved drugs (and some access to non-EMA-approved targeted and checkpoint-inhibitor therapies, as well as a limited number of clinical trials for rare TCs). However, this advantage is offset by diversity of approach. Parallel caretaking by family doctors and specialists in university and non-university settings and the absence of fixed patient paths for diagnosis and treatment result in substantial local, regional, and national variations in care. Personalised diagnostics are deployed in TC, and some recommendations exist, but in practice the choice of diagnostics depends on personal experience, access to diagnostics, and reimbursement for health care professionals. Personalised molecular diagnostics are also subject to recommendations, but again, in practice, the picture is very mixed, leading to too many diagnostics, incomplete diagnostics, overtreatment and non-specialist treatment, and underdiagnosis in rare and aggressive TCs.

In Italy, TC incidence rates are among the highest worldwide, with increasing heterogeneity of incidence rates discernible across distinct regions and areas in Italy [53]. There were historically high levels of overdiagnosis and there is a need to reduce thyroid gland examination practices in the asymptomatic general population, at national and regional levels. Genetic tests for familial cases are widely available and reimbursed, while tumor genetic assessment is limited to some specialised centres and financially supported with research funds. Tissue banks are not available, and the gathering of archival material is challenging. There is no standardisation among centres regarding the processing of the biological material and the assays used for the genetic analyses. In specialised centres, access to EMA-approved drugs and access to selective inhibitors in the context of clinical trials differs. Wider variations are perceptible in the treatment of advanced thyroid cancers across the regions [20].

The Netherlands has an effective register based on pathology, and centralisation of care is seen as the best way forward to improve care for rare diseases [54]. Reimbursement by the hospitals can lead to limited clinical use of advanced testing tools; somatic genetic testing is available, but mainly as a part of panels used for other malignant tumors. Therefore, overall patients get what is available, not what they may specifically need—so molecular diagnostics are underutilised and not uniformly utilised. Routine genetic testing is available and is reimbursed for patients with medullary TC to exclude hereditary cases. Routine diagnosis in incidentally identified nodules, such as in MRI, is not recommended. Networks have been set up for patient care and research with a view to delivering uniform protocols, quality checks, transparency, and uniform information provision. Collaboration with patient networks has helped to improve doctor–patient communication (through organised talks with the patient, shared decision instruments and training, and guidance to address empowerment) and has also improved the organisation of patient care. 

Poland has been discouraging ultrasound scanning, but with uneven success. Attempts are made to overcome regional and local diversity by requiring contact with a certified expert centre prior to surgery. Access to and reimbursement of targeted treatment and of molecular profiling is an issue. Anaplastic TC is the most challenging for up-to-date management, and metastatic medullary TC is often treated by endocrinologists rather than by oncologists. 

In Spain, none of the regions currently supports molecular diagnostics for sporadic TC, except for inherited RET-mutated MTC. For advanced DTC, molecular diagnostics are performed in some hospitals but without protocols. The country suffers from inadequate data collection and classification to discern pathologies precisely. There is a lack of national registries for thyroid cancer patients, which makes it difficult to estimate the number of new cases per year and the percentage of patients that become refractory to RAI therapy. A Spanish Task Force for Thyroid Cancer on behalf of the Spanish Society of Endocrinology Thyroid Cancer Working Group (GTSEEN) and the Spanish Group for Orphan and Infrequent Tumours (GETHI) is aiming to establish a consensus on patient management [55]. Patient associations have been active participants in national medical meetings in recent years, to improve awareness, networking, promote a national registry, and improve quality of life after surgery or living with TC.

In Portugal, there are regional registries for cancer that include TC statistics. TC was the third cause of cancer in women, according to 2012 Portugal North Regional Cancer Registries [56]. Molecular diagnosis is not widely established in healthcare centres, but is supported by the national health system in selected cases for therapy decisions (RET in MTC and BRAF in ATC) or in suspicion of familial cases (MTC). Targeted therapies are usually available as second line in general hospitals and oncology centres. Endocrinologists have a pivotal role in the treatment and follow-up of patients with TC. A Thyroid Study Group networks under the auspices of the Portuguese Society of Diabetes, Endocrinology and Metabolism (GET-SPEDM), and a general hospital and research centres are part of the ENDO-ERN participating in the Main Thematic Group (MTGs) in thyroid cancer [57].

## 4. Discussion

### 4.1. How the EU Beating Cancer Plan (EBCP) Could Help

It should support state of the art diagnostics and treatment throughout Europe and create strong platforms for cross-border collaboration within Europe and beyond, also covering registries and data and biobanking, and facilitating access to (e-)health records. In clinical trials, a structural financing framework for long-term aims could enable strategic planning instead of solitary calls that support opportunistic strategies, both enabling collaboration with the pharmaceutical industry and actively supporting academic research. The plan should crucially contribute to facilitating equitable reimbursement and reduce unnecessary regulatory and administrative burdens. This would benefit clinical trials, interoperability, cross-border collaboration, sustainability, and cost effectiveness. In addition, it should ensure a legislative and General Data Protection Regulation (GDPR) framework to facilitate and encourage cross-border collaborations. The EBCP can have direct benefits if properly applied. For citizens, it could avoid ultrasound thyroid screening in asymptomatic individuals, leading to reduced incidence, as citizens are not classed as patients. Active surveillance for cured patients could mean less intense but more personalised follow-up strategies, meaning that they need not be patients forever. For clinicians, it could reduce unnecessary treatments in terms of total thyroidectomies and RAI if guidelines [28,29] and recommendations are endorsed. At a wider level, precision medicine could accurately monitor populations and take preventive initiatives with a focus on relevant high-risk populations identified (Table 3). 

### 4.2. Recommendations

The approach to TC needs a rethink to early and correct identification of patients and improve their patient journey and treatment outcomes, including improved quality of life. 

All patients should be referred to comprehensive cancer centres. Such an approach would instate an optimal patient management mitigating overdiagnosis and overtreatment and would improve access to biomarker testing, clinical trials, and appropriate systemic therapies for patients with advanced forms of TC. It would also help establish focused national expertise on rare TC cases, promote research, training, awareness, and facilitate international collaboration.

Treatment and molecular testing guidelines should be regularly updated at regional and national levels to facilitate the reimbursement and availability for the most effective diagnostics and therapies. In the most recent update of the clinical practice guidelines, ESMO highlights for the first time the need to test for actionable cancer alterations in thyroid cancer. It is essential that national guidelines are coordinated with the European and international ones [29].

Quality comprehensive genomic testing should be incorporated in clinical practice (tissue or liquid samples) for all TC patients that can benefit from targeted therapies. In order to facilitate correct diagnosis, pathology practice, data collection and analysis, as well as communication with the treating physicians should be improved, with the provision of registers based on pathology. National cancer registries should distinguish specific diseases with standardised, quality-controlled systems, including details of case management and including long-term survival data. National and international data bases are needed not only for refractory thyroid cancer, but for all types of TC. 

The challenge of reconciling data exchange with data protection must be met.

Thyroid gland examination practices in the asymptomatic general population need updating.

Ongoing monitoring of DALYS and mortality rates is required to ensure true disease is not being missed as treatment becomes more selective.

Certified institutions should be employed for testing.

More clinical trials with new drugs and diagnostics are needed, including multicentre prospective trials for ATC patients, and EU funding should focus on prospective rather than retrospective studies. 

With the rapid growth of big data analytics in genomics, imaging, and clinical outcomes, health systems should make it possible to generate a personalised, data-driven risk profile to customise therapy for individual patients, aiming for both long-term tumour control and manageable adverse effects.

There is a need for a general change in philosophy so that TC is managed in the same way as for any other cancer: evidence-based medicine using results from prospective trials. The EU should sponsor prospective academic trials to avoid the risk that financial support is wastefully absorbed by ineffective retrospective work that fails to promote the improvements needed in medical practice. Such trials are feasible and can change medical practice—such as by avoiding the use of radioiodine in at least two thirds of TC patients. Moreover, it is crucial to allocate more funding to clinical research and this is something that should be addressed at the European level (Table 3).

## 5. Conclusions

The EU should cooperate to identify patients who can benefit from targeted treatment, by supporting projects aimed at developing personalised treatments and the underlying infrastructure. TC needs more outreach to rare patients to combine and cooperate on cases, and to fund advanced testing and research. There is a need for a common EU policy on molecular diagnostics for advanced DTC that is systematic and standardised as to when and how it should be used.

Closer and systematic collaboration is required among healthcare professionals, through multidisciplinary teams that can ensure high standards in diagnosis and treatment. It is necessary to educate physicians in working across disciplines and promote a corresponding change in attitudes. European Reference Networks are already demonstrating their potential in this area and the current review of their evolution should reinforce their capacity [57,58].

Multidisciplinary tumor boards should be established as a matter of routine, to assist in analysis of molecular diagnostics and provision of expert treatment advice.

Cooperation must be improved between cancer networks and scientific societies on an international level and between all national networks, and specialised networks set up in all European countries.

Patients should be engaged more closely in treatment decisions, and education and training are needed to raise TC awareness and information in the general population. Empowerment and education of patients and a boost to health literacy is also required. In addition, patient participation in the decision-making process is needed, relating to their diagnosis and treatment, the organisation of patient care (local, national, cross-border), the organisation and execution of scientific research, and the preparation of information material.

## Figures and Tables

**Table 1:** Challenges regarding tackling thyroid cancer, categorised as demand side and supply side issues. The methodology used to form a consensus during the EAPM panel.

The Challenges Outlined by the EAPM Panelists	
Demand Side	Supply Side
The lack of governance;	Reimbursement is patchy, uncertain, and inadequate across the EU;
International guidelines are not completely concordant;	The testing methodology and the infrastructure for conducting and validating tests is uneven at EU level;
An adequate clinical trials framework (and appropriate funding) does not exist	Guidelines mainly focus on clinically actionable biomarkers with approved therapies;
Clinical standardisation is not sufficient;	NGS, as a technology to detect genomic alterations, is not yet routine in clinical practice;
Awareness of TC diagnosis and management is low;	Insufficient provision of molecular profiling and precision medicine-based selection for patients with aggressive tumors;
Public awareness is very low;	Insufficient provision of multidisciplinary forums to ensure collaboration among the wide range of skills involved;
Lack of knowledge on thyroid cancer;	Patient access to appropriate and standardised diagnostic tests, therapies, and expertise varies across Europe;

**Table 2:** Some national perspectives in tackling TC emerged from the EAPM panel.

Country	Perspective
Denmark	- preoperative assessment of thyroid nodules remains a diagnostic challenge; - scintigraphy and ultrasound are performed as part of the investigation;
France	- has one of the highest incidence rates in Europe; - the magnitude of overdiagnosis increased rapidly and varied substantially across regions; - Vivre sans Thyroïde (patient forum)—ensures close cooperation with doctors, associations, and umbrella organisations;
Germany	- cases have increased in part due to a rise in well-differentiated PTC; - a good cost-free access of all patients to all levels of TC care; - personalised diagnostics are deployed in TC;
Italy	- increased heterogeneity of incidence rates discernible across distinct regions and areas; - a need to reduce thyroid gland examination practices in the asymptomatic general population; - genetic tests for familial cases are widely available and reimbursed;
Netherlands	- has an effective register based on pathology; - routine genetic testing is available and is reimbursed for patients with medullary TC; - collaboration with patient networks has helped to improve doctor–patient communication;
Poland	- access to and reimbursement of targeted treatment and of molecular profiling is an issue; - anaplastic TC is the most challenging for up-to-date management;
Spain	- none of the regions currently supports molecular diagnostics for sporadic TC; - there is an inadequate data collection and classification to discern pathologies precisely; - patients associations have been active participants in national medical meetings in recent years;
Portugal	- regional registries for cancer that include TC statistics; - molecular diagnosis is not widely established in healthcare centres; - targeted therapies are usually available as second line in general hospitals and oncology centres.

**Table 3:** Summarised conclusions and recommendations regarding tackling TC across Europe.

Conclusions and Recommendations Emerged from the EAPM Panel	
Conclusions	Recommendations
At a wider level, precision medicine could accurately monitor populations and take preventive initiatives;	Ensure that national guidelines are coordinated with the European and international ones;
With the rapid growth of big data, health systems have a chance to generate a personalised, data-driven risk profiles to customise therapy for individual patients;	Create a strong platforms for cross-border collaboration within Europe and beyond, also covering registries and data and biobanking;
Cooperation in the EU to identify patients who can benefit from targeted treatment is not yet fully incorporated;	Establish focused national expertise on rare TC cases, promote research, training, awareness, and facilitate international collaboration;
There is a need for a systematic EU policy on molecular diagnostics for advanced DTC;	Update treatment and molecular testing guidelines at regional and national levels;
Collaboration among healthcare professionals through multidisciplinary teams is still not fully in force;	Improve data collection and analysis, as well as communication with the treating physicians;
There is a need to educate physicians in working across disciplines and promote a corresponding change in attitudes;	Update of thyroid gland examination practices in the asymptomatic general population;
There is a lack of multidisciplinary tumor boards;	Incorporate quality comprehensive genomic testing in clinical practice;
Cooperation between cancer networks and scientific societies on an international level and between all national networks must be improved;	National cancer registries should distinguish specific diseases with standardised, quality-controlled systems;
Patients are not engaged enough in treatment decisions;	All patients should be referred to comprehensive cancer centres;
Empowerment and education of patients is needed;	National and international data bases are needed for all types of TC;
The EBCP has a chance to crucially contribute to facilitating equitable reimbursement and reduce unnecessary regulatory and administrative burdens.	Funding and collaboration opportunities in the EBCP, Cancer Mission, and other funding should be investigated actively.

## Data Availability

Not applicable.

## References

[1] NHS (2022). Thyroid cancer, Diagnosis [WWW Document]. https://www.nhs.uk/conditions/thyroid-cancer/diagnosis/.

[2] American Cancer Society (2021). What is Thyroid Cancer [WWW Document]. Who. http://www.cancer.org/cancer/thyroidcancer/detailedguide/thyroid-cancer-what-is-thyroid-cancer.

[3] Lim H., Devesa S.S., Sosa J.A., Check D., Kitahara C.M. (2017). Trends in Thyroid Cancer Incidence and Mortality in the United States, 1974–2013. JAMA-J. Am. Med. Assoc..

[4] Smittenaar C.R., Petersen K.A., Stewart K., Moitt N. (2016). Cancer incidence and mortality projections in the UK until 2035. Br. J. Cancer.

[5] Sung H., Ferlay J., Siegel R.L., Laversanne M., Soerjomataram I., Jemal A., Bray F. (2021). Global Cancer Statistics 2020: GLOBOCAN Estimates of Incidence and Mortality Worldwide for 36 Cancers in 185 Countries. CA Cancer J. Clin..

[6] American Cancer Society (2022). Key Statistics for Thyroid Cancer [WWW Document]. American Cancer Society. https://www.cancer.org/cancer/thyroid-cancer/about/key-statistics.html.

[7] Pizzato M., Li M., Vignat J., Laversanne M., Singh D., La Vecchia C., Vaccarella S. (2022). The epidemiological landscape of thyroid cancer worldwide: GLOBOCAN estimates for incidence and mortality rates in 2020. Lancet Diabetes Endocrinol..

[8] American Cancer Society (2022). Thyroid Cancer Survival Rates, by Type and Stage [WWW Document]. https://www.cancer.org/cancer/thyroid-cancer/detection-diagnosis-staging/survival-rates.html.

[9] Khosravi M.H., Kouhi A., Saeedi M., Bagherihagh A., Amirzade-Iranaq M.H. (2017). Thyroid Cancers: Considerations, Classifications, and Managements. Diagnosis and Management of Head and Neck Cancer.

[10] Schlumberger M., Leboulleux S. (2020). Current practice in patients with differentiated thyroid cancer. Nat. Rev. Endocrinol..

[11] Haroon Al Rasheed M.R.H., Xu B. (2019). Molecular Alterations in Thyroid Carcinoma. Surg. Pathol. Clin..

[12] Nylén C., Mechera R., Maréchal-Ross I., Tsang V., Chou A., Gill A.J., Clifton-Bligh R.J., Robinson B.G., Sywak M.S., Sidhu S.B. (2020). Molecular Markers Guiding Thyroid Cancer Management. Cancers.

[13] Horgan D., Borisch B., Richer E., Bernini C., Kalra D., Lawler M., Ciliberto G., Van Poppel H., Paradiso A., Riegman P. (2020). Propelling Health Care into the Twenties. Biomed. Hub.

[14] Vaccarella S., Franceschi S., Bray F., Wild C.P., Plummer M., Maso L.D. (2016). Worldwide Thyroid-Cancer Epidemic? The Increasing Impact of Overdiagnosis. New Engl. J. Med..

[15] Tuttle R.M. (2018). Controversial Issues in Thyroid Cancer Management. J. Nucl. Med..

[16] Jegerlehner S., Bulliard J.-L., Aujesky D., Rodondi N., Germann S., Konzelmann I., Chiolero A., Mousavi M., Camey B., Bouchardy C. (2017). Overdiagnosis and overtreatment of thyroid cancer: A population-based temporal trend study. PLoS ONE.

[17] Bates M.F., Long K.L., Sippel R.S. (2017). Ensuring Quality in Thyroid Cancer Surgery. US Endocrinol..

[18] Campennì A., Barbaro D., Guzzo M., Capoccetti F., Giovanella L. (2020). Personalized management of differentiated thyroid cancer in real life–practical guidance from a multidisciplinary panel of experts. Endocrine.

[19] Ito Y., Onoda N., Okamoto T. (2020). The revised clinical practice guidelines on the management of thyroid tumors by the Japan Associations of Endocrine Surgeons: Core questions and recommendations for treatments of thyroid cancer. Endocr. J..

[20] Pierannunzio D., Fedeli U., Francisci S., De Paoli A., Toffolutti F., Serraino D., Zoppini G., Borsatti E., Di Felice E., Falcini F. (2022). Thyroidectomies in Italy: A population-based national analysis from 2001 to 2018. Thyroid.

[21] Krajewska J., Kukulska A., Oczko-Wojciechowska M., Kotecka-Blicharz A., Drosik-Rutowicz K., Haras-Gil M., Jarzab B., Handkiewicz-Junak D. (2020). Early Diagnosis of Low-Risk Papillary Thyroid Cancer Results Rather in Overtreatment Than a Better Survival. Front. Endocrinol..

[22] Aliyev E., Ladra-González M.J., Sánchez-Ares M., Abdulkader-Nallib I., Piso-Neira M., Carnero M.G.R., Vieiro-Balo P., Pérez-Becerra R., Gude-Sampedro F., Barreiro-Morandeira F. (2020). Thyroid Papillary Microtumor: Validation of the (updated) porto proposal assessing sex hormone receptor expression and mutational BRAF gene status. Am. J. Surg. Pathol..

[23] Siderova M. (2019). Thyroid Cancer: Diagnosis, Treatment and Follow-Up. https://www.intechopen.com/chapters/61460.

[24] Wang L., Ganly I. (2017). Post-treatment surveillance of thyroid cancer. Eur. J. Surg. Oncol..

[25] Drozdovitch V. (2021). Radiation Exposure to the Thyroid After the Chernobyl Accident. Front. Endocrinol..

[26] Pacini F., Capezzone M., Elisei R., Ceccarelli C., Taddei D., Pinchera A. (2002). Diagnostic 131-Iodine Whole-Body Scan May Be Avoided in Thyroid Cancer Patients Who Have Undetectable Stimulated Serum Tg Levels After Initial Treatment. J. Clin. Endocrinol. Metab..

[27] Takamura N., Orita M., Saenko V., Yamashita S., Nagataki S., Demidchik Y. (2016). Radiation and risk of thyroid cancer: Fukushima and Chernobyl. Lancet Diabetes Endocrinol..

[28] Filetti S., Durante C., Hartl D., Leboulleux S., Locati L., Newbold K., Papotti M., Berruti A. (2019). Thyroid cancer: ESMO Clinical Practice Guidelines for diagnosis, treatment and follow-up. Ann. Oncol..

[29] Filetti S., Durante C., Hartl D., Leboulleux S., Locati L., Newbold K., Papotti M., Berruti A. (2022). ESMO Clinical Practice Guideline update on the use of systemic therapy in advanced thyroid cancer. Ann. Oncol..

[30] ETA (2022). ETA Guidelines [WWW Document]. https://www.eurothyroid.com/guidelines/eta_guidelines.html.

[31] Pešorda M., Kuna S.K., Huić D., Herceg D., Despot M., Samardžić T., Gnjidić M., Belev B. (2020). Kinase Inhibitors in the Treatment of Thyroid Cancer: Institutional Experience. Acta Clin. Croat..

[32] Ragusa F., Ferrari S.M., Elia G., Paparo S.R., Balestri E., Botrini C., Patrizio A., Mazzi V., Guglielmi G., Foddis R. (2022). Combination Strategies Involving Immune Checkpoint Inhibitors and Tyrosine Kinase or BRAF Inhibitors in Aggressive Thyroid Cancer. Int. J. Mol. Sci..

[33] Nickel B., Semsarian C., Moynihan R., Barratt A., Jordan S., McLeod D., Brito J.P., McCaffery K. (2019). Public perceptions of changing the terminology for low-risk thyroid cancer: A qualitative focus group study. BMJ Open.

[34] Tahara M. (2018). Management of recurrent or metastatic thyroid cancer. ESMO Open.

[35] Accardo G., Conzo G., Esposito D., Gambardella C., Mazzella M., Castaldo F., Di Donna C., Polistena A., Avenia N., Colantuoni V. (2017). Genetics of medullary thyroid cancer: An overview. Int. J. Surg..

[36] Acquaviva G., Visani M., Repaci A., Rhoden K.J., De Biase D., Pession A., Giovanni T. (2017). Molecular pathology of thyroid tumours of follicular cells: A review of genetic alterations and their clinicopathological relevance. Histopathology.

[37] Agrawal N., Akbani R., Aksoy B.A., Ally A., Arachchi H., Asa S.L., Auman J.T., Balasundaram M., Balu S., Baylin S.B. (2014). Integrated Genomic Characterization of Papillary Thyroid Carcinoma. Cell.

[38] Bonhomme B., Godbert Y., Perot G., Al Ghuzlan A., Bardet S., Belleannée G., Crinière L., Do Cao C., Fouilloux G., Guyetant S. (2017). Molecular Pathology of Anaplastic Thyroid Carcinomas: A Retrospective Study of 144 Cases. Thyroid.

[39] Cocco E., Scaltriti M., Drilon A. (2018). NTRK fusion-positive cancers and TRK inhibitor therapy. Nat. Rev. Clin. Oncol..

[40] Higgins M.J., Forastiere A., Marur S. (2009). New directions in the systemic treatment of metastatic thyroid cancer. Oncology.

[41] Song Y.S., Park Y.J. (2019). Genomic Characterization of Differentiated Thyroid Carcinoma. Endocrinol. Metab..

[42] Lin J.S., Bowles E.J.A., Williams S.B., Morrison C.C. (2017). Screening for thyroid cancer: Updated evidence report and systematic review for the US preventive services task force. JAMA-J. Am. Med. Assoc..

[43] Hall S.F., Irish J., Groome P., Griffiths R. (2014). Access, excess, and overdiagnosis: The case for thyroid cancer. Cancer Med..

[44] Horgan D. (2018). From here to 2025: Personalised medicine and healthcare for an immediate future. J. Cancer Policy.

[45] Horgan D., Ciliberto G., Conte P., Baldwin D., Seijo L., Montuenga L.M., Paz-Ares L., Garassino M., Penault-Llorca F., Galli F. (2020). Bringing Greater Accuracy to Europe’s Healthcare Systems: The Unexploited Potential of Biomarker Testing in Oncology. Biomed. Hub.

[46] de la Fouchardiere C., Fugazzola L., Taylor J., Appetecchia M., Besic N., Bongiovanni A., Buffet C., Costante G., Gay S., Grande E. (2021). 1750P Molecular genotyping in refractory thyroid cancers: Results of a European survey. Ann. Oncol..

[47] Locati L., Colombo E., Dedecjus M., de la Fouchardière C., Bongiovanni M., Netea-Maier R. (2021). 1754P Current picture of Anaplastic Thyroid Cancer patients’ care and meetable needs: A survey of 94 institutions from the EORTC Endocrine and Head and Neck Cancer Groups. Ann. Oncol..

[48] Gram S.B., Rasmussen J.H., Feldt-Rasmussen U., Bentzen J., Lelkaitis G., von Buchwald C., Hahn C.H. (2019). Risk of Thyroid Cancer in 1,504 Patients Referred for Thyroid Surgery with Assumed Benign Histology. Eur. Thyroid J..

[49] Leenhardt L., Grosclaude P., Chérié-Challine L. (2004). Increased incidence of thyroid carcinoma in france: A true epidemic or thyroid nodule management effects? Report from the french thyroid cancer committee. Thyroid..

[50] Li W., Justice-Clark T., Cohen M.B. (2021). The utility of ThyroSeq ^®^ in the management of indeterminate thyroid nodules by fine-needle aspiration. Cytopathology.

[51] (2022). Vivre Sans Thyroïde. https://www.forum-thyroide.net/.

[52] Radespiel-Tröger M., Batzler W.U., Holleczek B., Luttmann S., Pritzkuleit R., Stabenow R., Urbschat I., Zeissig S.R., Meyer M. (2014). Rising incidence of papillary thyroid carcinoma in Germany. Bundesgesundheitsblatt. Gesund-Heitsforschung Gesundh..

[53] Dal Maso L., Panato C., Franceschi S., Serraino D., Buzzoni C., Busco S., Ferretti S., Torrisi A., Falcini F., Zorzi M. (2018). The impact of overdiagnosis on thyroid cancer epidemic in Italy, 1998–2012. Eur. J. Cancer.

[54] van Dijk D., van Dijk B.A.C., Weistra A., Links T.P., Plukker J.T.M. (2020). Surgical Complications and Referral Patterns in 567 Patients with Differentiated Thyroid Cancer in the Northern Region of the Netherlands: A Population-Based Study To-wards Clinical Management Implementation. Ann. Surg. Oncol..

[55] Grande E., Sandi J.S., Capdevila J., Gonzalez E.N., Llopis C.Z., Asensio T.R.Y.C., Saez J.M.G., Jiménez-Fonseca P., Riesco-Eizaguirre G., Galofre J.C. (2015). Consensus on management of advanced medullary thyroid carcinoma on behalf of the Working Group of Thyroid Cancer of the Spanish Society of Endocrinology (SEEN) and the Spanish Task Force Group for Orphan and Infrequent Tumors (GETHI). Clin. Transl. Oncol..

[56] RORENO (2012). [WWW Document]. https://www.ipoporto.pt/dev/wp-content/uploads/2019/03/registo-oncol%C3%B3gico-norte.pdf.

[57] Endo-ERN (2022). Introduction [WWW Document]. https://endo-ern.eu/specific-expertise/thyroid/introduction/.

[58] De Vries F., Bruin M., Cersosimo A., Van Beuzekom C.N., Ahmed S.F., Peeters R.P., Biermasz N.R., Hiort O., Pereira A.M. (2020). An overview of clinical activities in Endo-ERN: The need for alignment of future network criteria. Eur. J. Endocrinol..

